# Patient‐reported outcomes after anterior cruciate ligament reconstruction with the JewelACL synthetic ligament: Multicenter study of at least 9 years follow‐up

**DOI:** 10.1002/jeo2.70838

**Published:** 2026-08-10

**Authors:** Klaudiusz Kosowski, Ewa Bręborowicz, Marek Olek, Maciej Wiśniewski

**Affiliations:** ^1^ Med Polonia Private Hospital Poznan Poland; ^2^ Traumatology, Orthopaedics and Hand Surgery Department Institute Poznan University of Medical Sciences Poznan Poland; ^3^ Traumatology and Orthopaedics Department District State Hospital Kępno Kępno Poland

**Keywords:** ACL reconstruction, functional scales, JewelACL, long‐term outcomes, synthetic ligament

## Abstract

**Purpose:**

Synthetic grafts for anterior cruciate ligament (ACL) reconstruction aim to address limitations of biological grafts, such as donor‐site morbidity and delayed recovery. JewelACL is a synthetic scaffold developed to enhance biological integration and mechanical durability. This multicenter study evaluated long‐term patient‐reported outcomes, adverse events, re‐rupture rates and return‐to‐sport outcomes following ACL reconstruction using JewelACL, with a minimum follow‐up of 9 years.

**Methods:**

A retrospective cohort of 58 patients who underwent ACL reconstruction between 2010 and 2014, using either JewelACL alone (*n* = 44) or JewelACL combined with hamstring autograft (hybrid technique; *n* = 14), was assessed. Patient‐reported outcome measures (PROMs), including the International Knee Documentation Committee (IKDC), Lysholm and Tegner scores, as well as complication and re‐rupture rates, were analysed. Long‐term follow‐up was based on telephone‐administered PROMs; no standardised clinical laxity testing or imaging (magnetic resonance imaging or x‐ray) was performed.

**Results:**

At a mean follow‐up of 10.8 ± 0.9 years, the re‐rupture rate was (5.2%) 3 cases out of 58. Median Lysholm and IKDC scores were 100 (interquartile range [IQR] 91–100) and 95 (IQR 89–99), respectively, indicating excellent long‐term knee function. The mean Tegner score at follow‐up was 5.2 ± 1.7, representing a modest decline from the pre‐injury level of 6.5, consistent with age‐related reductions in activity. No patient‐reported infections or hospital admissions related to the operated knee were reported. IKDC scores were significantly higher in the JewelACL‐only group than in the hybrid group (*p* = 0.02), while Lysholm and Tegner scores did not differ significantly.

**Conclusions:**

ACL reconstruction using JewelACL was associated with excellent long‐term PROMs, low re‐rupture rates and high return‐to‐sport rates. Although these findings are encouraging, interpretation is limited by the retrospective design, substantial loss to follow‐up and the absence of imaging or objective clinical assessments. There was no statistically significant difference in return‐to‐sport rates between the JewelACL group (85%) and the hybrid group (86%) (*p* = 1.00).

**Level of Evidence:**

Level IV.

**Abbreviations**
ACL: anterior cruciate ligament
BTB: bone‐tendon‐bone
IFU: instruction for use
IKDC: International Knee Documentation Committee
IQR: interquartile range
JewelACL: Brand name of a synthetic anterior cruciate ligament (ACL) implant
kGy: kilogray—gamma‐irradiation dose
LAD: ligament augmentation device
LARS: ligament advanced reinforcement system
PEEK: biocompatible thermoplastic polymer
PROMS: patient‐
  reported outcome measures
ROM: range of motion

## INTRODUCTION

Anterior cruciate ligament (ACL) reconstruction is one of the most commonly performed orthopaedic procedures worldwide. Approximately 200,000 ACL reconstructions are performed annually in the United States and Europe [25]. The primary aim of ACL reconstruction is to restore knee joint stability and functional biomechanics, enabling patients, particularly active individuals, to return to their previous level of physical activity. Over the years, significant advancements have been made in graft selection, tunnel positioning, fixation techniques and postoperative rehabilitation protocols, resulting in improved predictability of clinical outcomes [8, 14].

ACL injuries are most prevalent among young athletes, though the incidence in middle‐aged adults has also risen with the increasing popularity of recreational sports [8, 18].

Autografts, such as the bone‐patellar tendon‐bone graft and hamstring tendons (semitendinosus and gracilis), are commonly used in primary ACL reconstruction. Despite the improvements in surgical technique and rehabilitation, unsatisfactory results, defined by a limited range of motion (ROM), persistent pain or recurrent instability, still occur in approximately 3%–15% of patients [12]. In particular, the failure to achieve a stable ROM between 10° and 120° of knee flexion is considered clinically inadequate and can hinder return to sport or even daily activities. Compared with a synthetic graft, autograft reconstruction may be associated with donor‐site pain, variable or inadequate tissue quality (e.g., insufficient graft length or strength), sensory disturbance and scarring at the harvest site, longer operative time due to graft harvesting, potential impairment of knee flexor strength after hamstring harvest and a longer rehabilitation period.

In recent decades, synthetic ligaments have been investigated as either standalone grafts or scaffolds to support the ingrowth of biological tissue. The complex anatomical, biomechanical and proprioceptive characteristics of the native ACL have made it difficult to create a permanent synthetic substitute that replicates native function. Earlier synthetic ligaments, such as Gore‐Tex, Dacron, LAD and carbon fibre implants, exhibited high failure rates due to complications including synovitis, foreign body reactions and structural fatigue [18]. Some failures were due to material selection: carbon fibre and polytetrafluoroethylene. For instance, the Leeds‐Keio ligament, composed of polyester fibres, showed satisfactory results in approximately 80% of patients, though long‐term durability remained a concern [19]. The JewelACL (Xiros, UK) represents a more advanced synthetic scaffold designed to support biological integration and long‐term mechanical stability. Constructed from highly purified industrial polyester (polyethylene terephthalate), the implant features a tubular structure with longitudinal fibres offering tensile strength up to 1200 N and stiffness comparable to the semitendinosus tendon [24]. The open‐weave structure allows for fibroblast ingrowth, while dense transverse fibres permit secure fixation using widely accepted techniques, including cortical suspensory fixation (e.g., Endobutton, TightRope) and tibial interference screws made of titanium, PEEK or bioabsorbable materials.

Evaluations of the JewelACL device have predominantly reported outcomes at 2 years, leaving a significant gap in the literature concerning long‐term safety, durability and functional recovery [22]. This multicenter study evaluated long‐term patient‐reported outcomes, adverse events, re‐rupture rates and return‐to‐sport outcomes following ACL reconstruction using JewelACL, with a minimum follow‐up of 9 years.

## MATERIALS AND METHODS

### Study design and sample

This work is an observational, retrospective, three‐centre study. The study includes patients treated between October 2010 and December 2014 across multiple surgical centres (Med‐Polonia Hospital Poznań, Department of Traumatology and Orthopaedics District Hospital Turek and Department of Traumatology and Orthopaedics District Hospital Chodzież). It considers both total reconstructions and partial tissue‐sparing procedures. The study was conducted in accordance with the ethical principles of the Declaration of Helsinki and in full conformity with relevant regulations and in accordance with ISO 14155 Clinical investigation of medical devices for human subjects—Good Clinical Practice. The study clinical investigation plan was reviewed and approved by the appropriate independent ethics committee, the Wielkopolska Bioethical Commission (Date of approval: 17th June 2020). Patients who agreed to participate in the telephone follow‐up provided verbal consent, which was documented in the medical records.

Follow‐up assessments were conducted by telephone and were limited to PROM questionnaires and a structured complication/re‐rupture history. Objective clinical examination (e.g., Lachman/pivot shift/instrumented laxity) and radiologic assessment (x‐ray/magnetic resonance imaging [MRI]) were not performed as part of this follow‐up.

Eligible patients were adults (>18 years) who had undergone primary ACL reconstruction with the JewelACL device; patients were excluded if JewelACL was implanted for nonindicated conditions (per IFU) or in revision cases. Among 120 identified JewelACL surgeries performed consecutively between 1 October 2010 and 31 December 2014, 58 met the minimum 9‐year follow‐up criterion and formed the long‐term analysis cohort. Demographics and baseline characteristics are listed in Table 1.

**Table 1:** Demographics and baseline characteristics; 9‐year follow‐up information.

Variable	Category	All patients (*N* = 58)	JewelACL only (*N* = 44)	JewelACLhybrid (autograft) (*N* = 14)
Device	JewelACL only	44 (76%)		
	JewelACLhybrid	14 (24%)		
Age at surgery	—	31.8 ± 9.6	32.0 ± 9.6	31.3 ± 10.1
Gender	Female	10 (17%)	6 (14%)	4 (29%)
	Male	48 (83%)	38 (86%)	10 (71%)
Presurgery sports	No	3 (6%)	2 (6%)	1 (7%)
	Yes	55 (94%)	42 (94%)	13 (93%)
Cause of ACL failure	Sports injury	53 (91%)	41 (93%)	12 (86%)
	Traffic accident	2 (4%)	2 (5%)	0 (0%)
	Other accident	3 (5%)	1 (2%)	2 (14%)
Femoral fixation	Endobutton TightRope	1 (2%)	0 (0%)	1 (7%)
	Linvatec XO	0 (0%)	0 (0%)	0 (0%)
	Medgal screw	57 (98%)	44 (100%)	13 (93%)
	S&N EndoButton	0 (0%)	0 (0%)	0 (0%)
Tibial fixation	Medgal screw	58 (100%)	44 (100%)	14 (100%)

Abbreviation: ACL, anterior cruciate ligament.

### Clinical evaluation

At long‐term follow‐up (minimum 9 years), the study objectives were to document all recorded adverse events; evaluate patient‐reported outcomes using standardised, validated patient‐reported outcome measures (PROMs) that capture symptoms, function, activity and quality of life directly from patients without clinician interpretation (International Knee Documentation Committee [IKDC], Lysholm, Tegner); determine whether fixation type and concomitant autograft use influence outcomes; and quantify changes in PROM scores relative to earlier follow‐up time points. The IKDC Subjective Knee Evaluation Form assesses knee symptoms, function and sports activity on a 0–100 scale (higher scores indicate better function and fewer symptoms). The Lysholm score is an 8‐item questionnaire assessing pain, swelling, instability, locking, limping, stair climbing, squatting and need for support (0–100; higher is better), categorised as excellent (91–100), good (84–90), fair (65–83) and poor (<65). The Tegner Activity Scale is a self‐administered measure of activity level ranging from 0 to 10.

### Device description

**Figure 1:**
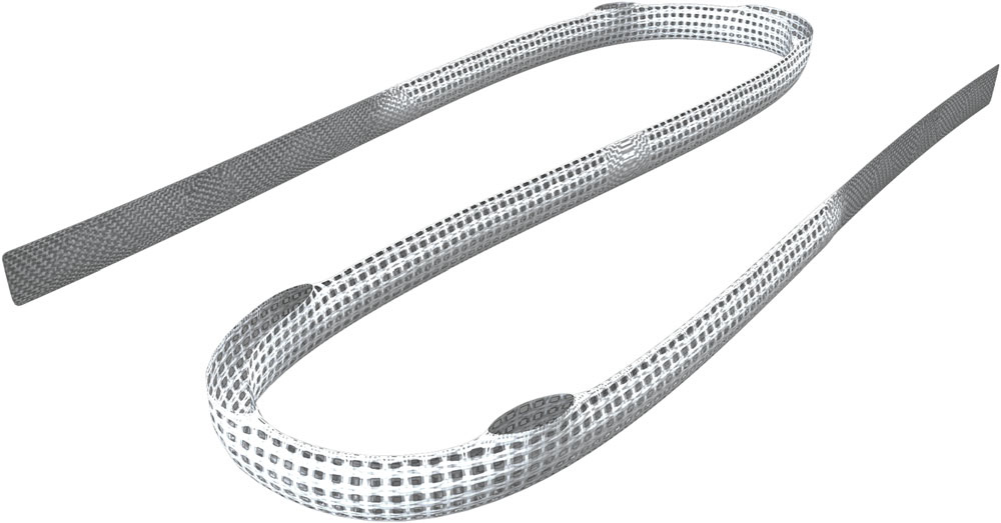
IFU library—Xiros Ltd., UK.

The JewelACL is a CE‐marked polyester scaffold for ACL reconstruction (Figure 1). It is matched in tensile strength to the semitendinosus hamstring tendon, which is typically 1200 N, and can be used with or without an additional tissue graft in either partial or total tissue‐sparing ACL reconstruction procedures, as described by the manufacturer [32].

The JewelACL is made from polyethylene terephthalate (polyester). The polyester has been subjected to a proprietary gas plasma process that modifies its surface properties, making it hydrophilic, without significantly altering the physical characteristics of the bulk material.

**Figure 2:**
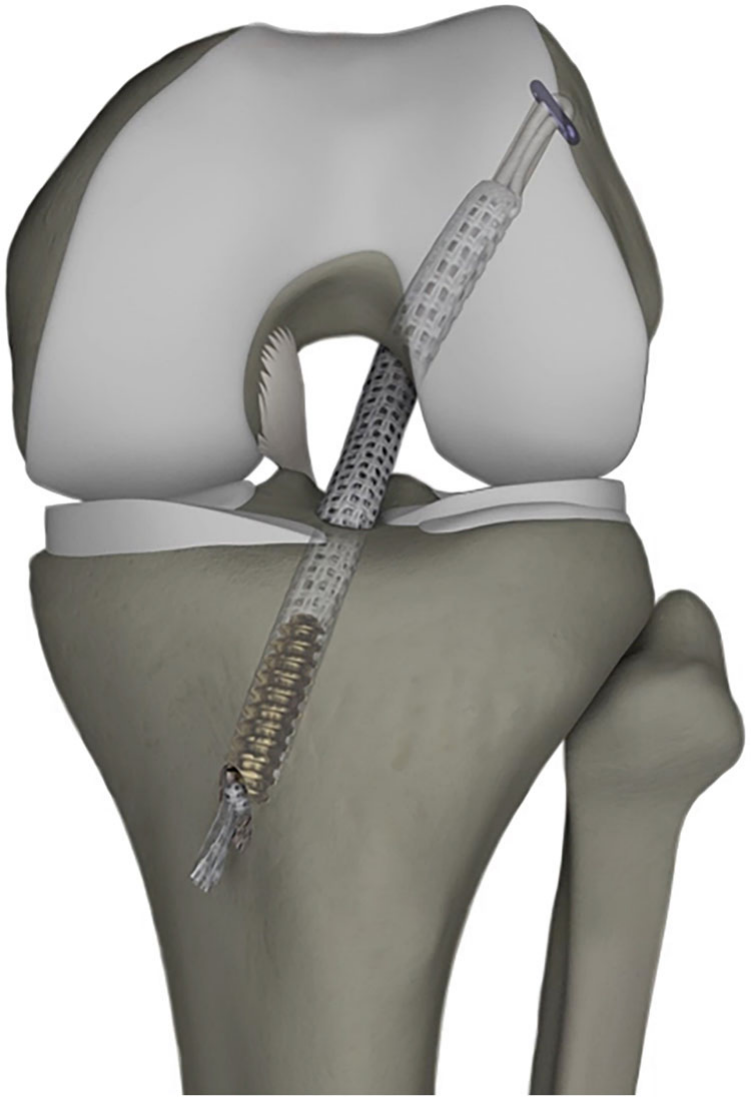
IFU library—Xiros Ltd., UK.

The polyester yarn is woven in a mock‐leno pattern, an open‐weave structure to permit the ingrowth of tissue, which can remodel to form a ‘neoligament′. It is tubular in structure, allowing the placement of a hamstring tendon graft (Figure 2).

The JewelACL is in the form of a tube 710 mm‐long with an internal diameter of 7 mm. The 7‐mm value is the nominal tubular size; when flattened, the scaffold′s effective width is governed by half its circumference, so it can be passed through smaller tunnels. It has a mean tensile strength of 1245 N (TR 105). When doubled, as in use, it will have a strength of up to 1977 N. The device does not incorporate medicinal substances, animal/human tissues or blood products and is supplied sterile (by gamma irradiation; 25–50 kGy).

### Surgical technique

JewelACL reconstruction is performed under general or spinal anaesthesia with the patient positioned supine on the operating table. A surgical tourniquet is applied. After standard skin preparation and draping, routine arthroscopic portals are created.

Following diagnostic arthroscopy, any chondral or meniscal lesions are treated. Using a femoral guide (Smith & Nephew or Arthrex) aimed at the anteromedial bundle (AMB) footprint (to ensure isometric placement of the graft), the femoral tunnel is established. A guidewire is passed, and the femoral tunnel is prepared according to the chosen fixation method. The tunnel is overdrilled to a diameter of 6 mm to accommodate the JewelACL graft, ensuring an adequate bony bridge remains distal to the graft.

**Figure 3:**
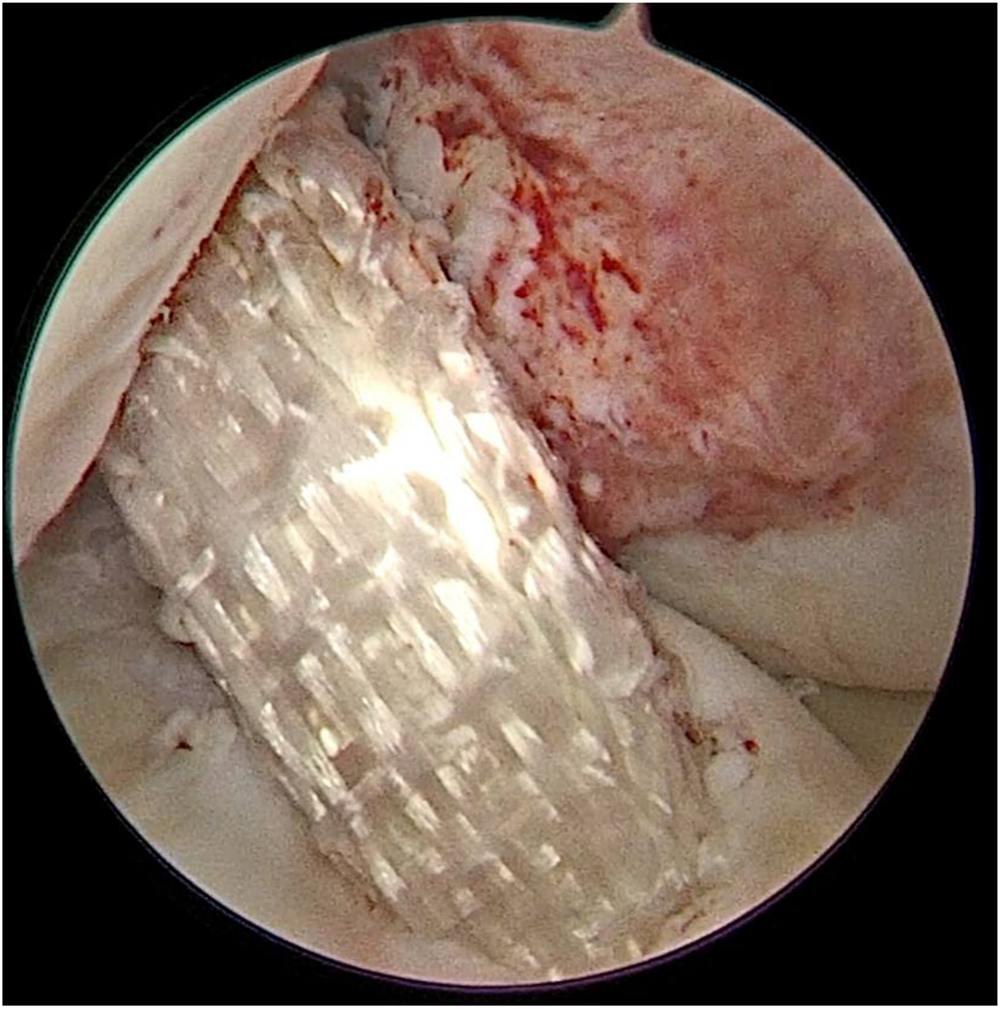
Arthroscopic view of the JewelACL in situ following fixation in the femoral and tibial tunnels. ACL, anterior cruciate ligament.

The tibial tunnel is then created using a tibial guide (also Smith & Nephew or Arthrex) directed at the AMB tibial footprint. This tunnel is also overdrilled to 6 mm using a guidewire. The synthetic JewelACL graft is passed through both tunnels. Femoral fixation is achieved either with cortical suspensory fixation (Endobutton/TightRope) or with interference screw fixation, depending on surgeon preference. Tibial fixation is completed with a titanium interference screw, which is at least 1 mm larger in diameter than the tibial tunnel to ensure secure fixation. Finally, the knee is cycled through full flexion and extension to properly tension the graft, with final tensioning performed according to the fixation construct used (Figure 3).

Although the JewelACL device can be secured using either cortical suspensory fixation (e.g., Endobutton/TightRope) and/or interference screws, fixation in this study was predominantly performed using Medgal interference screws (femoral: 57/58, tibial: 58/58). Suspensory femoral fixation (Endobutton/TightRope) was used in 1/58 cases. The fixation choice reflected surgeon preference and routine practice at the time of surgery.

In the case of a hybrid technique, the procedure is commenced by harvesting a gracilis or semitendinosus tendon. The tendon is then introduced into the tubular structure of the JewelACL graft through ‘windows’ provided in the Jewel graft. The technique for preparing the tunnels and graft fixation is identical to that used in the standalone JewelACL technique.

### Statistical methods

A single analysis was performed after completion of long‐term follow‐up data collection (≥9 years postprocedure). The full analysis population comprised all recruited patients with long‐term follow‐up; for each endpoint, patients with missing data were excluded only from that analysis, and all summaries and statistical tests were performed on the corresponding available‐case dataset. Continuous variables were summarised using the number of nonmissing observations and, when approximately normally distributed, the mean ± standard deviation; nonnormally distributed continuous variables were summarised using the number of observations, median and interquartile range. Categorical variables were summarised as counts and percentages based on the nonmissing denominator. The occurrence of complications was analysed as a binary outcome (yes/no) and reported as the number and percentage of patients with at least one complication, together with a 95% confidence interval (CI) for the proportion. PROM outcomes (IKDC, Lysholm, Tegner) were treated as continuous variables and compared between device/fixation groups using an unpaired *t*‐test when normally distributed and the Mann–Whitney test otherwise. Changes in PROMs between the long‐term assessment and earlier time points were evaluated using paired *t*‐tests for normally distributed differences (reporting mean change with 95% CI) or Wilcoxon matched‐pairs tests for nonnormally distributed differences (reporting median change with 95% CI). In addition to analyses of the overall cohort, descriptive summaries were also produced for the predefined subgroups of patients treated with JewelACL alone (*n* = 44) and JewelACL combined with autograft (*n* = 14). For all analyses, only observed data were used; missingness was assumed to be missing at random, and no imputation was performed.

## RESULTS

Follow‐up time from surgery was summarised for the overall cohort (*N* = 58) and by treatment subgroup. Mean ± SD follow‐up was 10.8 ± 0.9 years (range 9.0–12.6) in the full cohort, 10.8 ± 0.9 years (range 9.0–12.6) in the JewelACL‐only group (*N* = 44) and 10.8 ± 0.9 years (range 9.1–12.3) in the JewelACL + autograft (hybrid) group (*N* = 14). Overall, the mean follow‐up was approximately 11 years, and all patients had at least 9 years of follow‐up (minimum 9.0 years).

*Primary efficacy analysis*. Re‐rupture data (Table 2) were available for all 58 patients at long‐term follow‐up (≥ 9 years). Overall, three patients experienced a re‐rupture (5.2%), with a 95% CI indicating that the underlying proportion could be as high as 14.4%. All re‐ruptures occurred in the JewelACL‐only group (3/44; 6.8%), whereas no re‐ruptures were reported in the JewelACL + autograft (hybrid) group (0/14; 0.0%). Two of three re‐ruptures in the JewelACL‐only group occurred during football at approximately 10 years postsurgery (one revised using a semitendinosus/gracilis autograft), all were confirmed by MRI and one was associated with a slip on pavement at 9 years postsurgery.

**Table 2:** Occurrence of re‐ruptures.

Patient group	Re‐ruptures *n*/*N*	Percentage (95% confidence interval [CI])
All patients	3/58	5.2% (1.1%, 14.4%)
JewelACL only	3/44	6.8% (1.4%, 18.7%)
JewelACLhybrid	0/14	0.0% (0.0%, 23.2%)

*Note*: Between‐group comparison for JewelACL‐only versus JewelACL hybrid: *p* = 0.56, Fisher′s exact test. Interpretation is limited by the small number of events.

Abbreviation: ACL, anterior cruciate ligament.

Statistical comparisons of PROMs between the two device types suggested no evidence of a difference between types for either Lysholm or Tegner scores. However, there was evidence of a significant difference between the devices for the IKDC scores at the 10‐year follow‐up. For this outcome, scores were higher in the JewelACL‐only group, with a median of 95, than in the JewelACL hybrid group, where the median score was only 91 (Table 3).

**Table 3:** PROMs and sports participation (9‐year follow‐up).

Outcome/timepoint	All patients (*n*)	Summary	JewelACL only (*n*)	Summary	JewelACL hybrid (*n*)	Summary	*p‐*Values
Sports postsurgery	55	47 (85%)	41	35 (85%)	14	12 (86%)	NA
Lysholm follow‐up*	56	100 [91, 100]	42	100 [91, 100]	14	99 [84, 100]	0.16
Tegner pre‐injury	57	6.5 ± 1.8	43	6.6 ± 1.7	14	6.4 ± 2.0	NA
Tegner follow‐up*	56	5.2 ± 1.7	42	5.3 ± 1.8	14	5.0 ± 1.4	0.63
IKDC follow‐up*	56	95 [89,99]	42	95 [89,99]	14	91 [83,95]	0.02

Abbreviation: ACL, anterior cruciate ligament; IKDC, International Knee Documentation Committee; PROMs, patient‐reported outcome measures.

The complications assessed during telephone follow‐up were re‐rupture, infection and hospital admission related to the operated knee. There were three cases of re‐rupture. No patient‐reported infections or hospital admissions related to the operated knee were reported.

*Secondary efficacy analysis*. The first secondary outcome was the occurrence of adverse events/complications at long‐term follow‐up (≥9 years). Apart from re‐ruptures, no patient‐reported infections or hospital admissions related to the operated knee were reported in the assessed cohort. For these telephone‐assessed complications, the observed rate was 0.0%, with an upper 95% CI bound of 6.5% and was similarly 0.0% in both the JewelACL‐only and JewelACL + autograft (hybrid) groups. Because follow‐up was telephone‐based, objective complications such as synovitis, foreign‐body reaction, graft integration failure or tunnel/bony changes could not be assessed. Additional secondary outcomes included return to sport and PROMs; summaries for the overall cohort and by subgroup are presented in Table 3.

**Figure 4:**
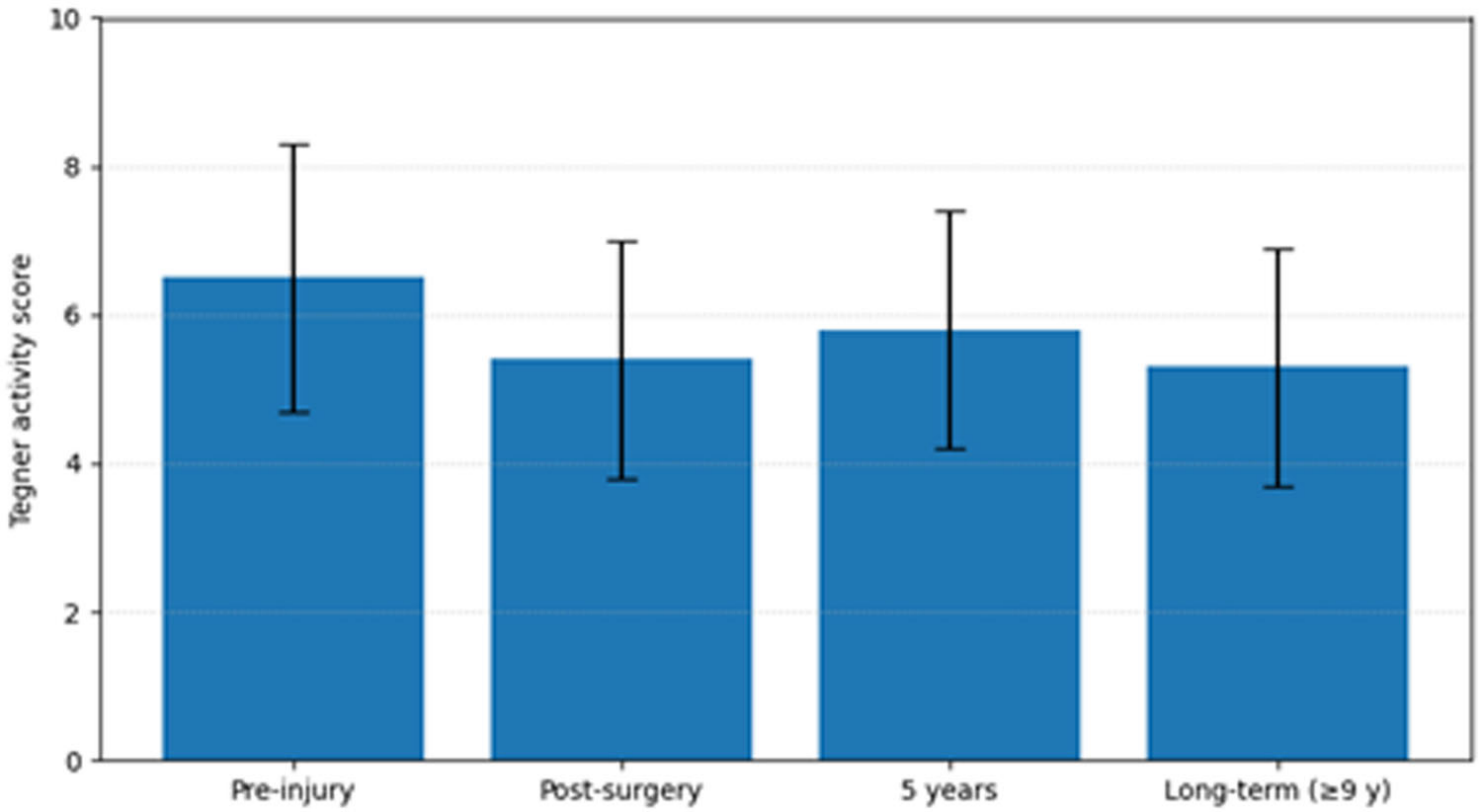
Changes in Tegner score (all patients, 9‐year follow‐up).

Summary statistics are reported as mean ± standard deviation, median [interquartile range] or number (percentage); (*) values are reported for patients who did not experience re‐rupture. Overall, 85% of patients participated in sports postsurgery, with similar rates in both groups. In the full cohort, the median Lysholm score at long‐term follow‐up was 100 [91, 100], the median IKDC score was 95 [89,99] and the mean Tegner score was 5.2 ± 1.7. Pre‐injury Tegner scores were available only for this outcome; therefore, changes in Tegner activity level from pre‐injury to the current follow‐up were analysed in the overall cohort. Figure 4 summarises the values at each time point, the mean change between time points (with 95% CI), and the corresponding *p*‐values for the within‐patient comparison.

The analyses indicated a significant reduction in Tegner scores from pre‐injury to long‐term follow‐up (≥9 years), with a mean decrease of 1.2 units (95% CI: −1.8 to −0.7; *p* < 0.001). Tegner scores remained stable from postsurgery to long‐term follow‐up (*p* = 0.64) and from 5 years to long‐term follow‐up (mean change −0.5, 95% CI: −1.0 to 0.1; *p* = 0.10).

*Subject accountability*. Table 4 summarises protocol deviations and subject accountability. At long‐term follow‐up (≥9 years), 62 patients were withdrawn or unavailable for assessment, leaving 58 patients (48% of the original 120) for analysis; complete datasets were available for 56 patients. Reasons for nonparticipation were loss to follow‐up (*n* = 44), declined participation (*n* = 13) and exclusion due to not meeting inclusion criteria (age <18 years at the time of surgery; *n* = 5). These patients were excluded from the final statistical analyses because the surgeries had already been performed as part of standard clinical care; their nonparticipation did not affect patient safety.

**Table 4:** Study protocol deviations.

Deviation	Site
Screen failures	0
Withdrawal criteria	62
Total withdrawn	62

## DISCUSSION

The most important findings of the present study are the low re‐rupture rate, favourable functional outcomes and high percentage of patients returning to sport [20]. The re‐rupture rate for all study participants receiving the JewelACL device for ACL reconstruction was 5.2% at a mean follow‐up of 10.8 ± 0.9 years (6.8% and 0.0% in the JewelACL only group and the JewelACL hybrid group, respectively). Rupture occurred between 9 and 10 years postsurgery, most commonly during football or after a slip injury.

Re‐rupture rates of between 0% and 30% at a minimum follow‐up of 2 years have been reported in state‐of‐the‐art literature for ACL reconstruction using autografts and allografts; [9, 10, 23] one study [31] reported a rate of 6.2% at more than 9 years follow‐up in 2700 implants. For the ligament advanced reinforcement system (LARS), a similar synthetic device to the JewelACL, re‐rupture rates of between 0% and 33% as reported in previous literature: [5] reported no patients requiring revision surgery in a 60‐patient cohort at a minimum of 5 years follow‐up; [25] reported a re‐rupture rate of 9.9% at a follow‐up of at least 5 years in a 71‐patient cohort; and [29] reported a re‐rupture rate of 33.3% at a median of 3.9 years in 55 patients.

The re‐rupture rate observed in this study, therefore, compares favourably with those reported in state‐of‐the‐art literature for alternative reconstruction methods and similar devices. Re‐ruptures can be sustained during subsequent sports trauma and therefore cannot necessarily be attributed to device insufficiency. Two re‐ruptures occurred while playing football, a known risk factor for primary ACL injury [30], and one occurred after a slip on the pavement at 9 years. No significant difference was detected between the JewelACL and the JewelACL hybrid groups regarding re‐rupture; however, this should be interpreted with caution, as the small number of re‐ruptures in this cohort limits statistical power.

Apart from re‐ruptures, no patient‐reported infections or hospital admissions related to the operated knee were identified at final follow‐up. Complications such as decreased ROM, persistent pain or need for subsequent revision surgery are recognised risks associated with ACL reconstruction. However, these events appear to be very rare with the JewelACL implant at final long‐term follow‐up (≥9 years).

The rate of participation in sport following reconstruction was 85% across all participants, consistent with previously reported figures of 56%–100% at a minimum 1‐year follow‐up for autograft and 86% to pre‐injury levels after 2 years for LARS [13, 15]. Before injury, 94% of patients participated in sports; however, not all previously active patients returned to sport. Given the retrospective nature of this study, there were no data on time to return to sport, as the study relied on participant recall.

There was a significant decrease in Tegner activity scores at follow‐up, suggesting that while patients returned to sport, they did not do so at their pre‐injury level. Postsurgery Tegner scores of between 4.3 and 9 have been reported for autografts [2, 6, 15, 17]; a mean Tegner score of 5.5 after 10 years has been reported [26]. For LARS, Tegner scores of between 5 and 8.6 have been reported [26]. Work presented in [13] reported a Tegner score of 7.5 after 2 years for a LARS hybrid with a hamstring autograft, and [7] noted scores of 6.0 after 10 years for LARS with remnant preservation. The mean follow‐up in the present study was 10.8 years, and the mean Tegner score was 5.2 ± 1.7 at this time point. This is comparable to scores reported in the state‐of‐the‐art literature but falls towards the lower end, particularly when compared with LARS ACL reconstruction [21]. Given that sports participation is expected to decline with age, Tegner scores may decrease with longer follow‐up, as observed in this study. However, this should be interpreted with caution, as younger patients may show increasing participation levels over time. In addition, patients often experience apprehension about returning to sport following reconstruction, which may affect Tegner scores despite the device functioning well [11].

The median IKDC score at final follow‐up was 95 [89,99], which compares well with scores reported for alternative reconstruction methods; mean scores of between 70 and 90 for ACL reconstruction using autografts and between 83 and 90 for reconstructions using LARS [1, 4, 6, 9, 15, 17]. The median Lysholm score at final follow‐up was 100 [91, 100], which again compares favourably with previously reported scores for alternative reconstruction methods; mean Lysholm scores of between 80 and 92 for autograft and between 87 and 98 for LARS [3, 4, 6, 15, 16, 26].

There was a significant difference in IKDC scores between the JewelACL only group and the JewelACL hybrid group at follow‐up, with a median IKDC of 95 [89,99] in the JewelACL only group compared to 91 [83,95] in the JewelACL hybrid group (*p* = 0.02). There was no significant difference in Lysholm scores between the groups (median 100 vs. 99, *p* = 0.16). While Tegner scores at follow‐up were slightly lower in the JewelACL hybrid group (5.0 ± 1.4) compared to the JewelACL only group (5.3 ± 1.8), this did not reach statistical significance (*p* = 0.63), suggesting there is no meaningful difference between the two in terms of function and level of sporting activity.

The participants in this study were predominantly male (83%), aged 18–58 years (mean 31.8 ± 9.6) and 94% of respondents participated in sports prior to injury. The target population for the JewelACL is musculoskeletally mature patients, and active patients are not contraindicated. The study population is therefore representative of the device′s target population.

This study provides limited data on women and older age groups (>60), and therefore, there is less confidence in the conclusions regarding the risk‐benefit profile for these groups. However, there is no reason to believe that the device would perform differently or that the risk‐benefit profile would be altered in these groups [27].

### Limitations

This study has several limitations that we list and briefly discuss in what follows:

An obvious flaw in this is that the participants were predominantly male, a potential limitation of which is the influence of sex on outcomes, as relatively few female patients were included and no sex‐based subgroup analysis was performed, in accordance with Sex and Gender Equity in Research guideline recommendations. However, this imbalance in the gender representations was by no means a matter of choice; it was determined by the patients presenting in our clinics.

Moreover, the number of total cases is relatively low, particularly in the JewelACL hybrid subgroup. Nearly half of the patients were unavailable for follow‐up for various reasons; some might have moved away without informing the hospital of their new address. We believe this is a common experience among orthopaedists, especially regarding long‐term follow‐up.

Additionally, regarding the lower number in the JewelACL hybrid group, this was driven by the confidence, gained at an early stage, in using the JewelACL as a standalone for ACL reconstruction.

Furthermore, the retrospective design, with telephone‐based follow‐up, precluded an objective assessment of complications such as synovitis or foreign‐body reactions. This is particularly important given the known complications associated with synthetic grafts, such as those associated with the LARS device [28].

Finally, because the present study focuses on a specific implant, potential commercial bias should be acknowledged.

## CONCLUSIONS

ACL reconstruction using JewelACL was associated with excellent long‐term PROMs, low re‐rupture rates and high return‐to‐sport rates. Although these findings are encouraging, interpretation is limited by the retrospective design, substantial loss to follow‐up and the absence of imaging or objective clinical assessments. There was no statistically significant difference in return‐to‐sport rates between the JewelACL group (85%) and the hybrid group (86%) (*p* = 1.00).

## AUTHOR CONTRIBUTIONS

**Klaudiusz Kosowski**: Conceptualisation; investigation; data curation; writing; writing review and editing; supervision. **Ewa Bręborowicz**: Data curation; writing; writing review and editing. **Marek Olek**: Investigation; writing; writing review and editing. **Maciej Wiśniewski**: Investigation; writing; writing review and editing. All authors have read and agreed to the published version of the manuscript.

## FUNDING INFORMATION

The authors have no funding to report.

## CONFLICT OF INTEREST STATEMENT

Klaudiusz Kosowski, <1>, serves as a Medical Consultant for Xiros Ltd, UK.

## ETHICS STATEMENT

Please include the name of the institutional review board (IRB) and the approval number. Greater Poland Medical Chamber, Poland, Bioethics Committee, Opinion No 181/2020 issued on the 17th of June 2020. The patient's consent statement was a part of the documents for the bioethics committee approval.

## Data Availability

The data that support the findings of this study are available from the corresponding author upon reasonable request. Picture 1 and Picture 2—taken from the IFU library with permission of Xiros Ltd., UK.
